# The effect of deep brain stimulation in Parkinson’s disease reflected in EEG microstates

**DOI:** 10.1038/s41531-023-00508-x

**Published:** 2023-04-17

**Authors:** Martin Lamoš, Martina Bočková, Sabina Goldemundová, Marek Baláž, Jan Chrastina, Ivan Rektor

**Affiliations:** 1 Brain and Mind Research Program, Central European Institute of Technology, Masaryk University, Brno, Czech Republic; 2 Movement Disorders Center, First Department of Neurology, Masaryk University School of Medicine, St. Anne’s Hospital, Brno, Czech Republic; 3 Department of Neurosurgery, Masaryk University School of Medicine, St. Anne’s Hospital, Brno, Czech Republic

**Keywords:** Biomarkers, Parkinson's disease

## Abstract

Mechanisms of deep brain stimulation (DBS) on cortical networks were explored mainly by fMRI. Advanced analysis of high-density EEG is a source of additional information and may provide clinically useful biomarkers. The presented study evaluates EEG microstates in Parkinson’s disease and the effect of DBS of the subthalamic nucleus (STN). The association between revealed spatiotemporal dynamics of brain networks and changes in oscillatory activity and clinical examination were assessed. Thirty-seven patients with Parkinson’s disease treated by STN-DBS underwent two sessions (OFF and ON stimulation conditions) of resting-state EEG. EEG microstates were analyzed in patient recordings and in a matched healthy control dataset. Microstate parameters were then compared across groups and were correlated with clinical and neuropsychological scores. Of the five revealed microstates, two differed between Parkinson’s disease patients and healthy controls. Another microstate differed between ON and OFF stimulation conditions in the patient group and restored parameters in the ON stimulation state toward to healthy values. The mean beta power of that microstate was the highest in patients during the OFF stimulation condition and the lowest in healthy controls; sources were localized mainly in the supplementary motor area. Changes in microstate parameters correlated with UPDRS and neuropsychological scores. Disease specific alterations in the spatiotemporal dynamics of large-scale brain networks can be described by EEG microstates. The approach can reveal changes reflecting the effect of DBS on PD motor symptoms as well as changes probably related to non-motor symptoms not influenced by DBS.

## Introduction

Deep brain stimulation (DBS) has been successfully used to treat various symptoms in several neurological and psychiatric disorders^1^. Advanced motor symptoms in Parkinson’s disease (PD) are the most common indication. Despite the general effectiveness of the DBS treatment, it has limitations. There are hardly predictable inter-individual differences in the responsiveness, and also adverse side effects can complicate the therapy, mainly dysarthria and neuropsychiatric complications^2^. The exact mechanism of DBS functioning and the cause of the side effects are still not fully understood, nor is the impact of DBS on whole brain functioning. The main target structure for the stimulation in PD is the subthalamic nucleus (STN), which is connected with thalamus, pallidum, and cortical regions through the basal ganglia-cortex circuit and hyperdirect pathway^3^. To better understand the mechanisms of DBS, recent research questions moved to study the effect of the stimulation on alterations in large-scale brain networks^4–7^.

Network changes are often explored by functional magnetic resonance imaging (fMRI), including in the context of DBS^6,8,9^. However, electroencephalography (EEG) is easily accessible and provides much higher temporal resolution^10^ to describe temporal dynamics. It has been shown that specific scalp-recorded neuronal oscillations are related to Parkinsonian symptoms and can be affected by DBS^11–13^. Moreover, with modern high-density scalp EEG systems (HDEEG), source space connectivity alterations induced by DBS reflect clinical responses to therapy^14^. For these reasons, the advanced analyses of surface EEG, especially HDEEG, have strong potential to provide clinically useful biomarkers.

Together with standard techniques oriented to describe variations in oscillatory patterns in particular brain areas, there is growing interest in the characterization of the spatiotemporal information of ongoing neural activity^15,16^. The EEG microstate concept lies in the representation of scalp EEG data with a sequence of quasi-stable prototypical maps, each lasting approximately 100 ms^17^. Many studies report that just a few dominant maps (around five) characterize the ongoing brain activity^15^ and can be related to large-scale brain networks usually described by fMRI^18–20^. While topographies of prototypical maps (microstates) are the same or very similar across subject groups and studies^15^, alterations in derived temporal parameters (duration, time coverage, occurrence, explained variance,…) of these microstates can be associated with the pathophysiology of various neurological diseases^21–24^ and neuropsychiatric diseases^25,26^.

Spatiotemporal dynamics analyzed by EEG microstates have already been explored in PD patients. Abnormal brain dynamics in temporal parameters were correlated with motor function and cognition in the early stages of PD in drug-free patients^27^. Another study^23^ presented differences in microstates between PD patients with and without dementia. The effect of dopaminergic treatment was also reported^28^. To our knowledge, only one study describes EEG microstates in patients treated by DBS;^29^ in that study, the explored effect of DBS was evaluated immediately in the first day of DBS treatment.

Characterizing brain states as an approach rich in quantifiable signatures may provide auxiliary markers for more accurate diagnosis and therapy in PD. Our present study aimed to identify EEG microstate changes in the context of chronic DBS treatment, to determine long-term impacts of the therapy in brain networks, and to explore associations with oscillatory activity and clinical scores.

## Results

**Fig. 1:**
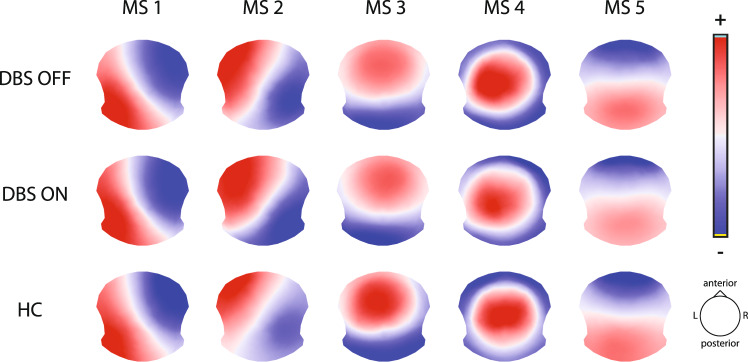
Topographies of five EEG microstates identified in each analyzed group. Red color indicates positive electric potential values, blue means negative. Note: the polarity in the topographies can be ignored (oscillations of the same neuronal generators).

Optimal number of cluster maps (EEG microstates) based on meta criterion was estimated to five in all analyzed groups and contained 81.9% of global explained variance (GEV) in the PD patient group during the DBS OFF state (further noted as DBS OFF), 83.5% in the PD patient group during the DBS ON state (DBS ON), and 84.7% in the healthy controls (HC). Each map (further referred as MS 1 – MS 5) showed highly similar topography across all three groups (Fig. 1).

**Fig. 2:**
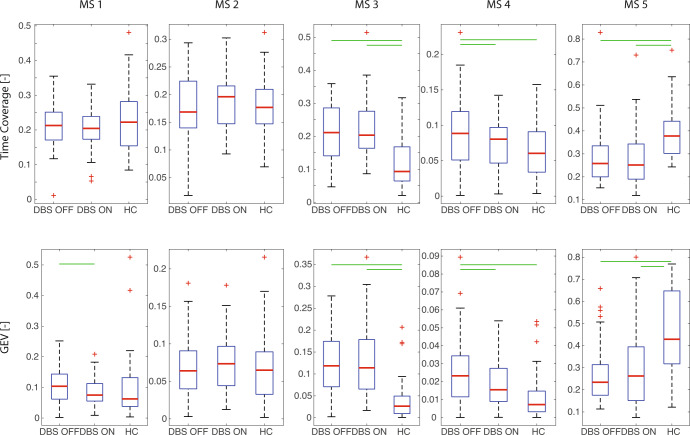
Comparison of temporal parameters (time coverage top, GEV bottom) of five identified microstates in each analyzed group. Each box covers the data from 25th to 75th percentiles; the red line in each box represents the median over subjects in a particular group, and whiskers represent 1.5 times the interquartile range (IQR). Red crosses show the outliers. Green lines mark significant differences (*p* < 0.05 FDR).

An analysis of the spatiotemporal properties of each microstate revealed statistically significant differences (*p* < 0.05 FDR) between groups in time coverage and GEV for MS 3, MS 4, and MS 5 (Fig. 2, Supplementary Table 2). Both parameters in MS 3 were significantly lower in the HC group. By contrast, in MS 5, the time coverage and GEV were significantly lower in the patient groups. In MS 4, both parameters showed significant differences between the DBS OFF and HC group and also between the DBS OFF and DBS ON group. Other computed microstate parameters (mean duration and occurrence) are shown in the supplementary materials (Supplementary Figure 3). Similar significant differences can also be observed for MS 3 and MS 5 in these parameters.

**Fig. 3:**
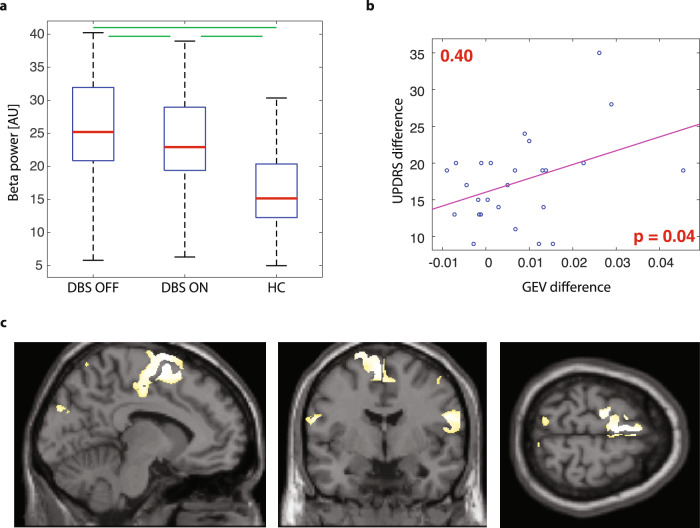
The significance of the EEG microstate 4. **a** - mean beta power during the presence of MS 4 compared across analyzed groups. Each box covers the data from 25th to 75th percentiles; the red line in each box represents the median over subjects in particular group, and whiskers represent 1.5 times the interquartile range (IQR). Red crosses show the outliers. Green lines mark significant differences (*p* < 0.05). **b** - correlation between GEV and MDS-UPDRS III score (differences between DBS OFF – DBS ON state). **c** - electrical source imaging of EEG segments where MS 4 was presented (10% highest activations).

Focusing on MS 4, the spectral analysis of EEG segments where MS 4 is dominantly presented revealed significant differences in beta power. Mean beta power was the highest in the DBS OFF group and the lowest in the HC group (Fig. 3a). Power changes in other frequency bands and other microstates are shown in the supplementary materials (Supplementary Fig. 5).

Independently of any microstate presence, the spectral analysis of the whole 5-minute session did not reveal any differences in the alpha and beta bands. DBS groups significantly differed from the HC group in delta and theta (Supplementary Figure 6 and 7).

To identify brain areas that are active during the presence of MS 4, we reconstructed these EEG segments into the source space (Fig. 3c). For MS 4, the largest active cluster was located over the supplementary motor area (SMA). Reconstructed sources of all microstates can be seen in Supplementary Figure 8.

Changes in the GEV of MS 4 between the DBS OFF and DBS ON groups were significantly correlated (*R* = 0.40, *p* = 0.04) with changes in the International Parkinson and Movement Disorders Society - Unified Parkinson’s Disease Rating Scale (MDS-UPDRS) III between the DBS OFF and DBS ON states (Fig. 3b). The parameters of MS 3 and MS 5 in the DBS ON group correlated with neuropsychological tests for executive functions and cognitive and emotional status (Table 1).

**Table 1:** Correlations between EEG microstate parameters and neuropsychological examination.

test	MS parameter		MS 1	MS 2	MS 3	MS 4	MS 5
DBS OFF							
Digit span	Time Coverage	*R* value	−0.15	−0.05	−0.23	−0.11	0.40*
		*p* value	0.47	0.80	0.26	0.60	0.04
	GEV	*R* value	−0.17	−0.16	−0.43*	−0.13	0.41*
		*p* value	0.41	0.45	0.03	0.54	0.04
DBS ON							
Mattis	Time Coverage	*R* value	0.06	−0.20	−0.40*	−0.06	0.49*
		*p* value	0.77	0.32	0.04	0.77	0.01
	GEV	*R* value	0.01	−0.16	−0.32	−0.09	0.29
		*p* value	0.98	0.43	0.11	0.67	0.15
MADRS	Time Coverage	*R* value	−0.35	−0.16	0.49*	−0.27	−0.28
		*p* value	0.08	0.45	0.01	0.18	0.16
	GEV	*R* value	−0.15	0.03	0.48*	−0.06	−0.32
		*p* value	0.45	0.87	0.01	0.77	0.11

*Mattis* Mattis dementia rating scale.

*MADRS* Montgomery-Åsberg Depression Rating Scale.

Star indicates significant correlations (*p* < 0.05 unc.).

## Discussion

Electrophysiological studies are crucial for the evolution of DBS therapy. Besides the huge importance of intracranial EEG and local field potentials (LFPs) analysis^30–32^, the scalp-recorded EEG studies are also currently within the main research focus. These have a potential to increase our knowledge of the exact DBS mechanisms of functioning on the whole brain level and to provide biomarkers for clinical practice^33^. PD is a heterogeneous disease with main motor symptoms of differing severity and a number of nonmotor symptoms. The responsiveness to different types of therapy is individual. Many symptom-specific markers have been described using the analysis of intracranial LFPs^34,35^ and these have been introduced into clinical practice for adaptive deep brain stimulation (aDBS)^36–38^. Such specific markers from scalp recordings are still largely unknown. Advanced analytical methods such as network connectivity measures, automated classifiers, and machine learning approaches offer significant promise for increasing this knowledge^39,40^. EEG microstate analysis is a methodological approach that reflects alterations in the spatiotemporal dynamics of large-scale brain networks^15,41^. We have found PD-specific patterns related to both motor and non-motor symptoms as well as the responsiveness to DBS therapy. Further studies are necessary, but microstate analysis seems to be a sensitive method that could be potentially helpful in tailoring individualized therapy in PD.

We have analyzed changes in a group of PD patients treated by DBS in the DBS OFF and DBS ON states as compared to matched HC. The five revealed microstates in all analyzed groups are similar to well-known topographies (left-right, right-left, anterior-posterior orientation, and frontocentral maximum)^15,16^.

In a comparison of temporal parameters (GEV and time coverage), two microstates (MS 3 and MS 5) can differentiate between PD and HC with no effect of DBS therapy. The same difference in the time coverage of the microstate with similar topography was already reported;^27^ the authors claimed that due to a correlation with MoCA (Montreal Cognitive Assessment) scores, this particular microstate can reflect the cognitive level of PD patients. This claim is in concordance with our results because the time coverage of MS 3 and MS 5 correlates with the Mattis scale, and MS 3 also correlates with the Montgomery-Åsberg Depression Rating Scale (MADRS) scale (see Table 1). The topography of that microstate (commonly referred to as “microstate C” in the literature) is often related to the activity of regions belonging to the salience and default mode network. Therefore, we suggest that changes in MS 3 and MS 5 are related to PN non-motor neuropsychiatric symptoms, mainly cognitive decline and depression, that are not influenced by DBS.

The DBS effect on GEV and time coverage parameters can be clearly seen in MS 4. No significant difference was found between HC and DBS ON, while DBS OFF differed from DBS ON and substantially from HC. The effect of DBS treatment on PD motor symptoms is therefore reflected only in MS 4. The topography of MS 4 is very similar to the commonly referred “microstate D”^15,16^. It is well known from clinical practice and other electrophysiological studies, that the effect of both dopaminergic treatment and DBS lead to similar clinical improvement of motor symptoms as well as oscillatory changes^30,42^. In our study, the influence of dopaminergic treatment cannot be evaluated as the patients were recorded after medicamental therapy withdrawal focusing on DBS. It has been documented in the available literature that the microstate D is also modified by levodopa intake in PD patients^27,28^. More studies using microstate analysis have been performed in psychiatric patients and the role of dopamine in microstate D parameters has been described in schizophrenia subjects as well^22,43^. Our work proves the involvement of MS 4 (microstate D) in motor-related functions influenced by DBS. Changes in GEV correlate to changes in MDS-UPDRS III scores between DBS OFF and DBS ON.

A study by Pal et al^23^. discussed the relation of motor areas and microstate D, suggesting it as a potential resting-state EEG biomarker of a Parkinsonian state. The topography of microstate D can be related to the activity of fronto-parietal (FP) areas^18,20^. The reconstruction of electrical sources from EEG segments where MS 4 is presented clearly localized the activity into the region of SMA, which plays a crucial role in the pathophysiology of PD and has connections with the STN and frontal circuits^44^.

The SMA is known to be functionally coupled to the STN, mainly in the beta frequency band^33,45^ and the beta activity between the STN and SMA is suppressed by DBS. Spectral analysis of our MS 4 segments shows the highest mean beta power in DBS OFF, significantly lower in DBS ON, and the lowest in HC group. Excessive synchronization of neural activity in the beta band represents a well-known pathophysiological mechanism in PD causing akinetic-rigid symptomatology^3,46,47^. DBS reduces beta synchronization in the sensorimotor network^48^, which improves motor symptoms. Thus, beta power changes during the presence of MS 4 across analyzed groups support the relation to motor functioning modulated by DBS.

An examination of the mean beta power from whole 5-minute segments (Supplementary Figure 6) did not reveal any changes among the analyzed groups in all 204 electrodes nor in 10 electrodes placed on the scalp over the supplementary motor areas. This indicates that the differences in scalp EEG in beta power related to motor symptoms are temporally and spatially constrained and can be described by changes in EEG microstates.

Recent research of acute post-operative DBS effect on brain dynamics^29^ found no changes in EEG microstates exclusively caused by DBS; however, according to the authors, the stable and obvious effect of DBS on brain networks is a result of long-term treatment influence and neural plasticity^5,29,45^.

Alterations in the spatiotemporal dynamics of large-scale brain networks can be described by EEG microstates. Applied to the high-density resting-state EEG in PD patients treated by DBS, it is possible to reveal microstates that relate to the non-motor symptoms of PD patients and are not influenced by DBS. In addition, MS4 (microstate D), so far linked to FP area activity and the attentional network, reflects the effect of DBS therapy. Its involvement in motor functioning is supported by changes in the oscillatory activity of the beta band, correlations with MDS-UPDRS III differences, and reconstructed neural sources dominantly localized within the supplementary motor area.

The presented results provide a different view of PD motor and non-motor symptoms and of how DBS influences large-scale brain network functioning. EEG microstates seem to be a sensitive and promising method reflecting PD-specific changes. Whether the microstate analysis could provide clinically useful biomarkers for DBS treatment remains to be clarified in further studies.

## Methods

### Subjects

Thirty-seven PD patients (mean age 61.3 ± 6.8 years, range 39–73 years, 9 females) with late motor complications (motor fluctuations, choreatic dyskinesias, and wearing-off phenomena) treated by STN-DBS (Medtronic Activa PC, St. Jude Medical Libra XP or Infinity stimulator) participated in the study. The subthalamic electrodes were implanted using a frame-based stereotactic technique (MRI-guided stereotaxy) preceded by finding the optimal location using intraoperative microelectrode recordings and stimulation. The duration of the DBS treatment lasted from 6 months to 8 years across subjects. The International Parkinson and Movement Disorders Society - Unified Parkinson’s Disease Rating Scale (MDS-UPDRS) III and a neuropsychological examination were used to evaluate each patient’s current clinical condition. Patients did not express signs of dementia or major depression and did not have any serious cognitive disorders according to previous detailed neuropsychological examination. For detailed patient characteristics and stimulation parameters, see Supplementary Table 1. A control group of thirty-seven healthy subjects (HC) was matched to the PD group in terms of age (mean age 60.3 ± 5.8 years, range 48–73 years; no significant difference with PD group, Wilcoxon test, *p* = 0.36) and sex (12 females; no significant difference with PD group, Chi-squared test, *p* = 0.44). The study was approved by the local ethics committee (The Research Ethics Committee, Masaryk University). All subjects were informed about the nature of the study and provided written informed consent form to take part in the study.

### EEG data acquisition

All subjects underwent resting-state recording by 256-channel scalp EEG (GES 400 MR, Electrical Geodesics, Inc.). The protocol contained one 5-minute session for the HC group and two 5-minute sessions for the PD group, which was measured after 12-hour medication withdrawal during the DBS OFF state and DBS ON state (in random order with a 30-minute pause between sessions). This protocol formed three groups for analysis, noted as DBS ON, DBS OFF, and HC. The sampling frequency was 1 kHz. HydroCel GSN 220MR cap with Cz reference electrode was used. Based on the EEG manufacturer’s recommendations, the impedances of all channels were held below 50kΩ.

### EEG preprocessing

The dataset was pre-processed in a standard manner for microstates analysis in the MATLAB R2017a environment (The MathWorks, Inc, Natick, USA) complemented by the EEGLAB toolbox^49^. The number of EEG channels was reduced to 204, discarding facial and neck-line electrodes. The ongoing 5-minute EEG data were filtered to 1–40 Hz by the second-order Butterworth filter in forward and reverse directions for zero phase distortion. Residues of DBS-related artifacts in the DBS ON data were visually detected in the frequency domain (several narrow peaks with substantially higher magnitude than background activity) and suppressed by a fast Fourier transform (FFT) filter (zeroing spectral lines on frequencies 29, 31, and 35 Hz). Details are shown in the supplementary material, Supplementary Figure 1. The same filtration was performed on the DBS OFF and HC data to maintain the same processing pipeline. Bad channels containing artifacts were detected automatically (when they were at least three standard deviations above the mean of all channels), checked by an experienced electroencephalographist, and interpolated by spheric spline (fewer than 10 channels; in DBS OFF group 4.0 ± 2.9, DBS ON group 4.1 ± 3.1, HC group 4.8 ± 2.2). Data segments with Global Field Power (GFP^50^,) more than ten standard deviations above the mean GFP were marked as artifacts (also checked by an electroencephalographist; 0.3% of the data in DBS OFF, 0.4% in DBS ON, and 0.3% in HC group). Independent component analysis (ICA) was used to suppress signals related to eye movements and electrocardiogram (ECG). After ICA decomposition, artifact-related components were identified manually by a visual inspection of their topography and time-series. EEG data were then back-reconstructed without these components. No more than four components were discarded. As a last preprocessing step, the data were downsampled to 125 Hz and re-referenced to the average. An example of the raw EEG compared to pre-processed data is shown in the supplementary material (Supplementary Figure 2).

### EEG microstates

Whole microstate analysis was computed in Cartool software^50^. The core of the approach is to find template maps, which represent the majority of the spatial data variability, to fit these templates back to the data, and finally to derive temporal parameters from data segments labeled by templates. Prototypical maps (microstates) can be revealed by a two-step clustering process. Cluster analysis was first applied to the EEG data of each subject and session individually only in the time points of GFP local maxima, where SNR is the highest. Epochs containing artifacts were skipped. Group-level clustering on the extracted subject-specific and session-specific cluster maps was then performed within each of three groups. The K-means technique was used in both steps. The optimal number of cluster maps was always defined based on six criteria (Gamma, Silhouettes, Davies and Bouldin, Point-Biserial, Dunn, Krzanowski-Lai Index), which were combined into the meta criterion as the median of all optimal numbers of clusters across all criteria^17,50^.

In each group, the optimal set of cluster maps selected by the meta criterion were fitted back to all samples of pre-processed EEG data except artifact-related epochs. Each data sample was labelled by the number of cluster map based on the highest spatial correlation (has to be higher than 0.5). To suppress small labelled segments, temporal smoothing with half window size 3 was used (see the supplementary material for details of the analysis).

### Four parameters of cluster maps (EEG microstates) were then derived

1. Global explained variance (GEV): global variance explained by the particular microstate.

2. Mean duration: average duration of the microstate continuous presence.

3. Time coverage: the portion of the analyzed time period during which the microstate is presented.

4. Occurrence: how often the microstate is presented per time interval.

Because of the not normal distribution in the parameter data (tested by Kolmogorov-Smirnov test), a two-sample nonparametric Wilcoxon test was calculated for each temporal parameter of each microstate between groups with FDR correction for multiple testing. For comparisons between the DBS ON and DBS OFF groups, a paired test was applied.

### EEG frequency analysis

Mean spectral power was calculated to describe differences in oscillations during the presence of each microstate. Power spectral density was evaluated in MATLAB using Fast Fourier Transform (FFT) with 0.1 Hz resolution. The mean power was estimated by trapezoidal numerical integration for each time point labelled with a particular microstate, frequency band of interest (1–4, 4–8, 8–13, 13–22, 22–35 Hz), and then averaged across electrodes.

To compare with a standard spectral EEG analysis, the mean power for the whole 5-minute session was also computed. Power spectral density was estimated for the same frequency bands and two variants of the selected scalp electrodes – all 204 preprocessed channels and 10 channels around the vertex (details in supplementary materials, Supplementary Figure 6).

Statistical comparisons were calculated similarly to comparisons of microstate temporal parameters. A two-sample nonparametric Wilcoxon test was used for each frequency band (standard spectral analysis) or each frequency band and each microstate (spectral analysis of microstates) between groups with FDR correction for multiple testing.

### Electrical source imaging

To uncover the neural generators of each microstate, a source reconstruction approach was used. The Montreal Neurological Institute (MNI) template was used for forward Locally Spherical Model with Anatomical Constraints (LSMAC) model construction and Local Auto-Regressive Averages (LAURA) was used for inversion. Six thousand solution points were equally distributed in a gray matter compartment of the head model. For each microstate, labelled time points were reconstructed into the source space, standardized to correct for the EEG power variability across time, and averaged across the time domain. Source maps of all microstates were converted to volumes and demeaned by subtracting the mean of all source maps from each source map to show microstate-specific sources.

### Limitations

The ICA procedure during the pre-processing was performed separately on each recording. Because of this, the selection of components with artifacts was very conservative to avoid suppression of relevant information in each subject group. The pipeline for electrical source imaging of scalp EEG data where particular microstates are presented uses the MNI template, not individual structural MRI data of each subject. This fact can slightly affect the precision of source localization^51^. The presence of DBS electrodes may also slightly affect the precision. Subcortical brain activity can still be detected from scalp EEG source imaging in these cases^52^. The group of our patients is heterogenous, with variable of DBS treatment durations (0.5 years to 8 years). On the other hand, we aimed to investigate the long-term effect of DBS and collect a large group of patients. Not all the patients can be included in the study because of artifacts from tremor or abnormal movements, so the data collection is challenging.

### Reporting summary

Further information on research design is available in the Nature Research Reporting Summary linked to this article.

## Supplementary information

Reporting Summary

Supplementary material

## Data Availability

The data that support the findings of this study are available from the corresponding author upon the request.
