# Supplementary material for: The effect of deep brain stimulation in Parkinson’s disease reflected in EEG microstates
Source: NPJ Parkinsons Dis. 2023 Apr 17;9:63. doi: 10.1038/s41531-023-00508-x (PMC10110608; doi:10.1038/s41531-023-00508-x)
Supplement: Supplementary file 1 — Reporting Summary [file 41531_2023_508_MOESM1_ESM.pdf]

nature portfolio

Corresponding author(s): Ivan Rektor

Last updated by author(s): Mar 27, 2023

Reporting Summary

Nature Portfolio wishes to improve the reproducibility of the work that we publish. This form provides structure for consistency and transparency in reporting. For further information on Nature Portfolio policies, see our Editorial Policies and the Editorial Policy Checklist.

Statistics

For all statistical analyses, confirm that the following items are present in the figure legend, table legend, main text, or Methods section.

n/a	Confirmed	
☐	☒	The exact sample size (*n*) for each experimental group/condition, given as a discrete number and unit of measurement
☐	☒	A statement on whether measurements were taken from distinct samples or whether the same sample was measured repeatedly
☐	☒	The statistical test(s) used AND whether they are one- or two-sided<br>*Only common tests should be described solely by name; describe more complex techniques in the Methods section.*
☐	☒	A description of all covariates tested
☐	☒	A description of any assumptions or corrections, such as tests of normality and adjustment for multiple comparisons
☐	☒	A full description of the statistical parameters including central tendency (e.g. means) or other basic estimates (e.g. regression coefficient) AND variation (e.g. standard deviation) or associated estimates of uncertainty (e.g. confidence intervals)
☐	☒	For null hypothesis testing, the test statistic (e.g. *F*, *t*, *r*) with confidence intervals, effect sizes, degrees of freedom and *P* value noted<br>*Give P values as exact values whenever suitable.*
☒	☐	For Bayesian analysis, information on the choice of priors and Markov chain Monte Carlo settings
☒	☐	For hierarchical and complex designs, identification of the appropriate level for tests and full reporting of outcomes
☐	☒	Estimates of effect sizes (e.g. Cohen's *d*, Pearson's *r*), indicating how they were calculated

Our web collection on statistics for biologists contains articles on many of the points above.

Software and code

Policy information about availability of computer code

Data collection	EGI Net Station - 5.3.0.1
Data analysis	MATLAB R2017a, EEGLAB 14.1.1, Cartool 3.91

For manuscripts utilizing custom algorithms or software that are central to the research but not yet described in published literature, software must be made available to editors and reviewers. We strongly encourage code deposition in a community repository (e.g. GitHub). See the Nature Portfolio guidelines for submitting code & software for further information.

Data

Policy information about availability of data

All manuscripts must include a data availability statement. This statement should provide the following information, where applicable:

- Accession codes, unique identifiers, or web links for publicly available datasets
- A description of any restrictions on data availability
- For clinical datasets or third party data, please ensure that the statement adheres to our policy

The data that support the findings of this study are available from the corresponding author upon the request.

Human research participants

Policy information about studies involving human research participants and Sex and Gender in Research.

Reporting on sex and gender	Both subject groups were matched in terms of age and sex. From 37 patients 9 of them were females, in healthy controls group from 37 subjects 12 of them were females.
Population characteristics	Parkinson's disease patients with late motor complications (motor fluctuations, choreatic dyskinesias, and wearing-off phenomena) treated by STN-DBS and control group of healthy subjects matched in terms of age and sex.
Recruitment	Patients: Movement Disorders Center, First Department of Neurology, Masaryk University School of Medicine, St. Anne's Hospital. Healthy controls: Core Facility MAFIL, Central European Institute of Technology, Masaryk University, Brno
Ethics oversight	The Research Ethics Committee, Masaryk University

Note that full information on the approval of the study protocol must also be provided in the manuscript.

Field-specific reporting

Please select the one below that is the best fit for your research. If you are not sure, read the appropriate sections before making your selection.

☒ Life sciences ☐ Behavioural & social sciences ☐ Ecological, evolutionary & environmental sciences

For a reference copy of the document with all sections, see nature.com/documents/nr-reporting-summary-flat.pdf

Life sciences study design

All studies must disclose on these points even when the disclosure is negative.

Sample size	Data collection of DBS patients is challenging. Not all the patients can be included in the study because of artifacts from tremor or abnormal movements. Sample size was chosen based on our previous studies where differencies in brain connectivity were exlored on similar sample size (N=34).
Data exclusions	artifacts from tremor or abnormal movements
Replication	Repeated calculations. Revealed EEG microstates are stable and reproducible.
Randomization	Not relevant, patient group contains all examined PD patients treated by STN-DBS, healthy controls group were age and sex matched.
Blinding	Blinding was not relevant for this study

Reporting for specific materials, systems and methods

We require information from authors about some types of materials, experimental systems and methods used in many studies. Here, indicate whether each material, system or method listed is relevant to your study. If you are not sure if a list item applies to your research, read the appropriate section before selecting a response.

Materials & experimental systems

n/a	Involved in the study	
☒	☐	Antibodies
☒	☐	Eukaryotic cell lines
☒	☐	Palaeontology and archaeology
☒	☐	Animals and other organisms
☒	☐	Clinical data
☒	☐	Dual use research of concern

Methods

n/a	Involved in the study	
☒	☐	ChIP-seq
☒	☐	Flow cytometry
☒	☐	MRI-based neuroimaging
