# Supplementary material for: The effect of deep brain stimulation in Parkinson’s disease reflected in EEG microstates
Source: NPJ Parkinsons Dis. 2023 Apr 17;9:63. doi: 10.1038/s41531-023-00508-x (PMC10110608; doi:10.1038/s41531-023-00508-x)
Supplement: Supplementary file 2 — Supplementary material [file 41531_2023_508_MOESM2_ESM.pdf]

Supplementary Table 1 – PD patient characteristics

No.	age	sex	DBS setting	medication	MDS – UPDRS III	
					off	on
**1**	65	F	2.5mA bilat./130Hz/91usec (Libra)	L-dopa, Ent, Rop	28	11
**2**	71	M	3.0V bilat./130Hz/ 90usec (Activa)	L-dopa, Rop	45	25
**3**	68	F	3.2V bilat/130Hz/90usec (Activa)	L-dopa, Ent, Rop	34	17
**4**	58	M	1.1mA bilat./130Hz/143usec (Libra)	L-dopa, Rop	40	26
**5**	52	M	3.3V bilat./130Hz/90usec (Activa)	L-dopa, Rot	42	14
**6**	55	M	1.5V bilat./130Hz/60usec (Activa)	L-dopa, Pra	46	29
**7**	64	F	2.4V bilat./130Hz/90usec (Activa)	L-dopa, Ent	50	17
**8**	61	F	2.4V bilat./130Hz/90usec (Activa)	L-dopa, Ent, Rop	35	15
**9**	39	M	3.9mA(bip)/3.0mA(bip)/130Hz/78usec (Libra)	L-dopa, Ent, Rop	48	13
**10**	60	M	1.9V bilat./130Hz/90usec (Activa)	L-dopa, Ent	31	18
**11**	65	F	3.2mA bilat. (bip)/130Hz/65usec (Libra)	L-dopa, Rop	44	31
**12**	53	M	2.2 V bilat./130Hz/90usec (Activa)	L-dopa, Ent, Rop	48	28
**13**	58	M	3.3V/4,3V/130Hz/90usec (Activa)	L-dopa, Rop, Rot, Bipe	53	37
**14**	55	F	1.4 mA/1,8mA/130Hz/91usec (Libra)	L-dopa, Ent, Pra	55	31
**15**	65	F	1.6V/1,4V/130Hz/90usec (Activa)	L-dopa, Pra	36	17
**16**	56	M	2.1V/2.1V(bip)/130Hz/90usec (Activa)	L-dopa, Ent	30	17
**17**	58	M	1.2V/2.5V/130Hz/90usec (Activa)	L-dopa, Ent, Rop	49	26
**18**	63	M	2.1V bilat./90usec/130Hz (Activa)	L-dopa, Ent, Rop, Sel	24	15
**19**	66	M	2.5V bilat./90usec/130Hz (Activa)	L-dopa, Ent, Rop	50	24
**20**	73	M	2.5V/ 3,0V /90usec/130Hz (Activa)	L-dopa, Ent	30	11
**21**	59	M	2.6V bilat/90usec/130Hz (Activa)	L-dopa	40	21
**22**	64	M	2.2V/90usec/130Hz (Activa)	L-dopa, Rop	38	14
**23**	66	M	2.8V/90usec/130Hz (Activa)	L-dopa, Pra	36	25
**24**	54	M	3.6V/60usec/130Hz (Activa)	L-dopa, Ent, Rop	36	17
**25**	65	M	1.4V/90usec/130Hz (Activa)	L-dopa, Pra	44	24
**26**	68	F	2.2mA/91usec/130Hz (Libra)	L-dopa, Ent, Ama	29	9
**27**	66	M	2.9V and 2.7V/90usec/130Hz (Activa)	L-dopa, Ent	36	21
**28**	54	M	3.2V/90usec/130Hz (Activa)	L-dopa, Ent, Rop, Ama	58	24

**29**	68	F	0.5 and 1.7V/90usec/130Hz (Activa)	L-dopa, Rop	42	23
**30**	64	M	2.6V/90usec/130Hz (Activa)	L-dopa, Ent, Rop	64	33
**31**	58	M	1.0V/90usec/180Hz (Activa)	L-dopa, Ent, Pra	26	17
**32**	69	M	3.3V/90usec/130Hz (Activa)	0	39	30
**33**	56	M	3V/60usec/130Hz (Activa)	L-dopa, Ent	36	21
**34**	66	M	1.5V and 4V (bip)/90usec/140Hz (Activa)	L-dopa, Tol, Rop, Ama	41	27
**35**	72	M	2.4 and 3.2(bip) mA/90usec/130Hz(Infinity)	L-dopa	38	21
**36**	55	M	3.1 and 2.6V/90usec/130Hz (Activa)	L-dopa, Pra	32	15
**37**	59	M	2.0(bip) and 2.4mA/90usec/130Hz (Infinity)	L-dopa, Ent, Pra	46	20

Bip - bipolar

Ama - amantadine, Bipe - biperiden, Ent - entacapone, L-dopa - levodopa, Pra - pramipexole, Rop - ropinirole, Rot- rotigotine, Sel - selegiline, Tol – tolcapone

EEG pre-processing

DBS artifacts

DBS device in DBS ON condition (mainly in unipolar stimulation setup) generates artifacts with substantial higher magnitude than EEG signals (here 130 Hz peak in frequency spectrum, Supplementary Figure 1B). Because of the great energy in the higher harmonics of 130Hz, the anti-aliasing filter of the amplifier is not able to suppress these components optimally and aliased frequencies contaminate the useful part of the EEG spectrum (several narrow peaks between approx. 30 and 110 Hz, Supplementary Figure 1B). Frequencies of peaks are subject specific. The standard filtration of the data for microstate analysis has a cut off usually at 40Hz. We observed residues of DBS artifacts (narrow peaks) at 29, 31 and 35Hz across the whole dataset under 40 Hz. Spectral lines on those frequencies were suppressed by zeroing in the spectral domain (addressed in the manuscript as the FFT filter). The zeroing of 29, 31, and 35 Hz spectral components was also performed in the DBS OFF and HC data. In Supplementary Figure 1A see also the detail of DBS artifacts in the time domain referring to the absence of saturation of amplifier by stimulation.

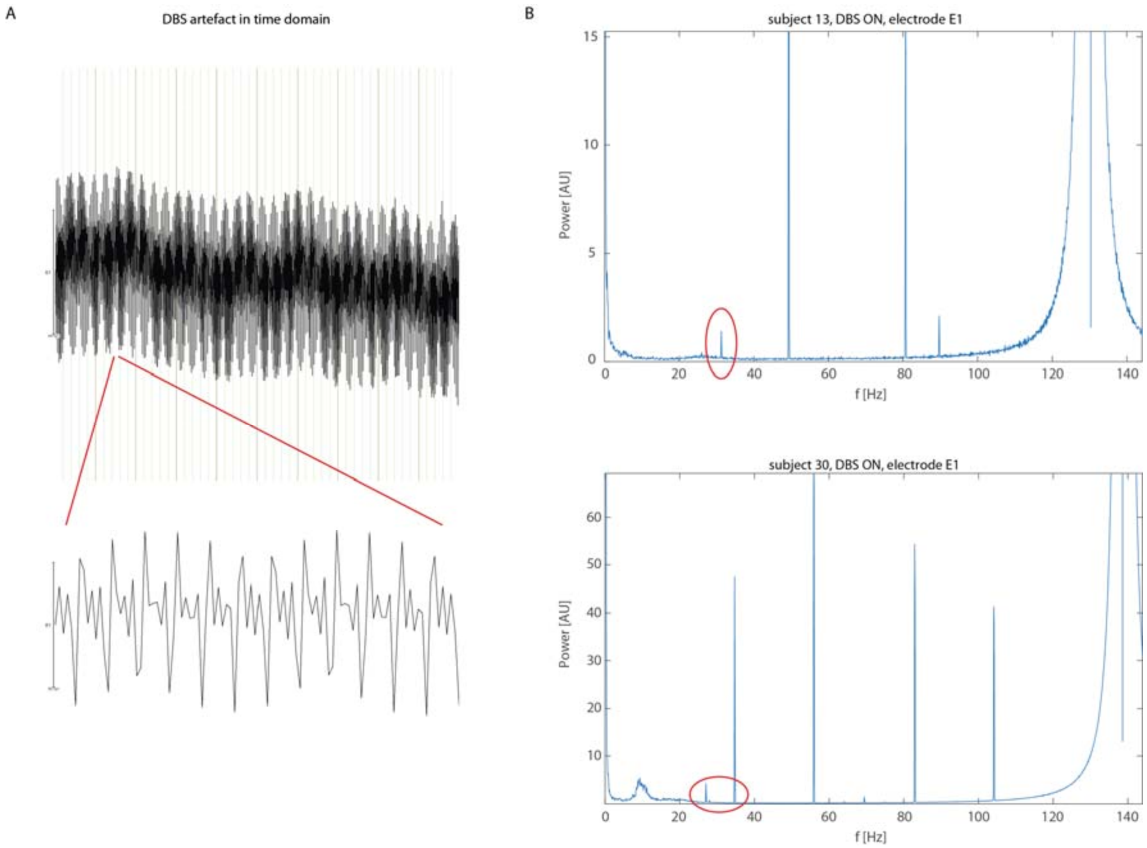

Supplementary Figure 1: Example of DBS artifacts in the time domain (A) and spectral domain (B). Red ellipses indicate subject-specific spectral lines under 40 Hz suppressed in each recording by zeroing in the frequency domain.

Comparison of raw and pre-processed data

A

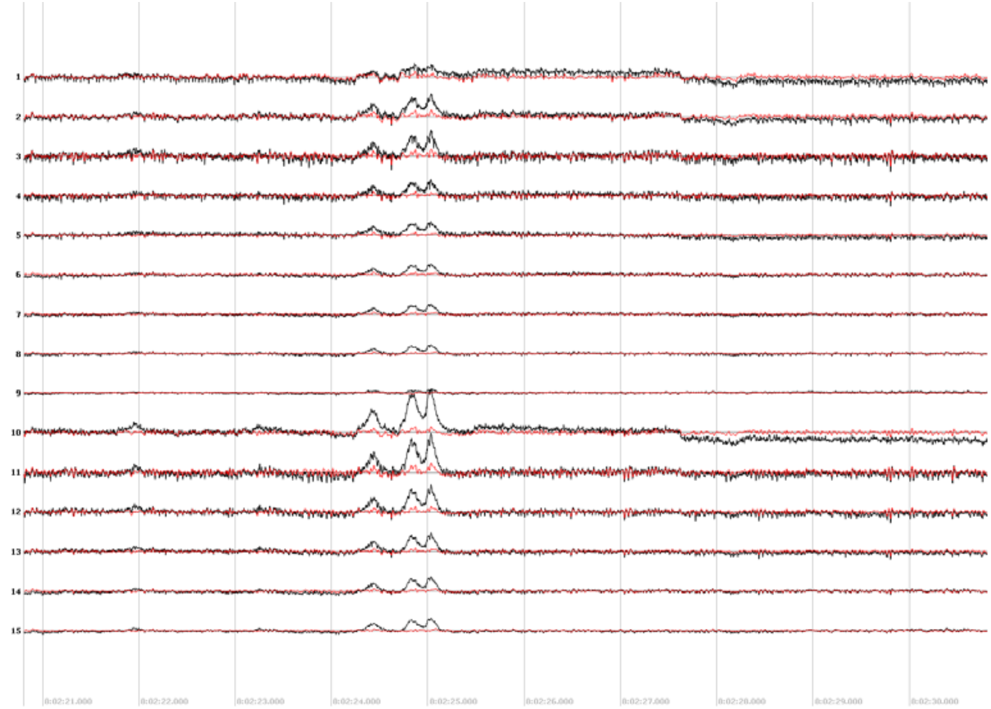

B

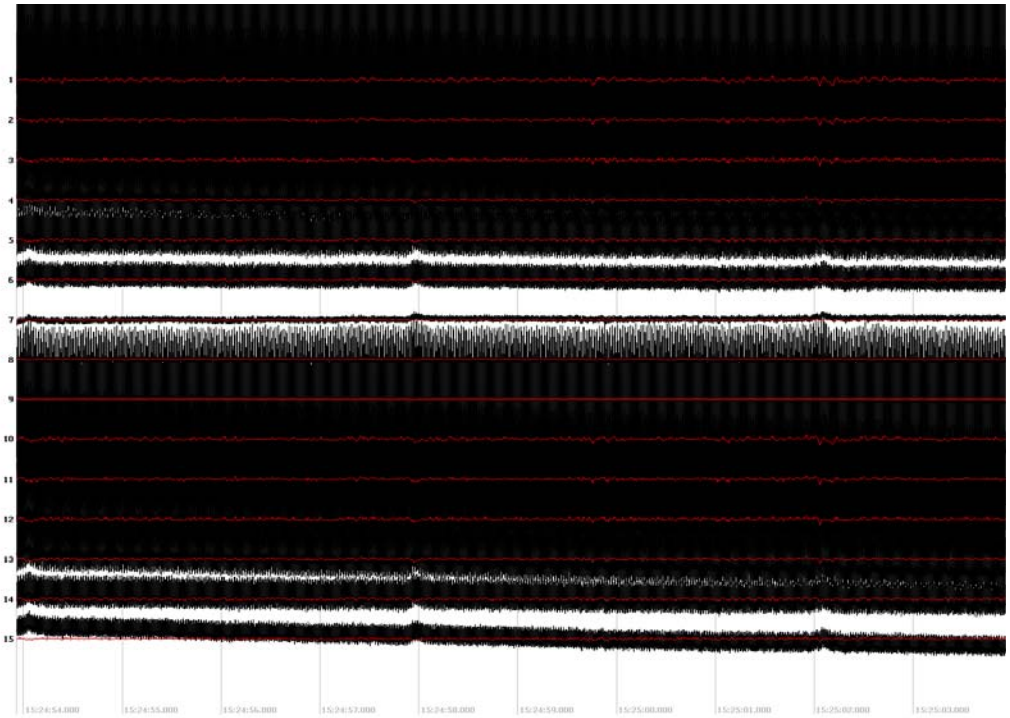

Supplementary Figure 2: Examples of raw and pre-processed data in the time domain. A – DBS OFF condition, B – DBS ON condition. Raw data in black, pre-processed data in red.

EEG microstates analysis

Cluster analysis parameters on subject-specific level (1st level): K-means, 100 randomizations, GFP local maxima time points used in the clustering.

Cluster analysis parameters on group level (2nd level): K-means, 200 randomizations, clustering of subject-specific template maps from 1st level.

The minimum correlation threshold for the samples to be assigned to a given cluster was 0.5. The polarity of the scalp potential field was ignored because the neuronal generators are the same regardless of the map polarity.

During the fitting procedure, a default setting of the Cartool software for processing continuous scalp EEG data was used. It also covers temporal postprocessing by temporal smoothing with half window size 3. It makes it possible to relabel very short segments lasting approx. 10ms.

Results

EEG microstates – temporal parameters (Time Coverage and GEV)

Supplementary Table 2: Results of statistical comparisons by nonparametric Wilcoxon tests. Significant differences are marked in red (p<0.05 FDR).

		p values		
		DBS OFF x DBS ON	DBS OFF x HC	DBS ON x HC
MS 1	Time coverage	0.1868	0.7385	0.3762
	GEV	0.0032	0.1480	0.7305
MS 2	Time coverage	0.0868	0.9577	0.2170
	GEV	0.5716	0.6073	0.3065
MS 3	Time coverage	0.0261	0.0000	0.0000
	GEV	0.2078	0.0000	0.0000
MS 4	Time coverage	0.0007	0.0046	0.1107
	GEV	0.0003	0.0000	0.0230
MS 5	Time coverage	0.2304	0.0000	0.0000
	GEV	0.1584	0.0000	0.0010

EEG microstates – effect of DBS treatment duration on time coverage and GEV parameters

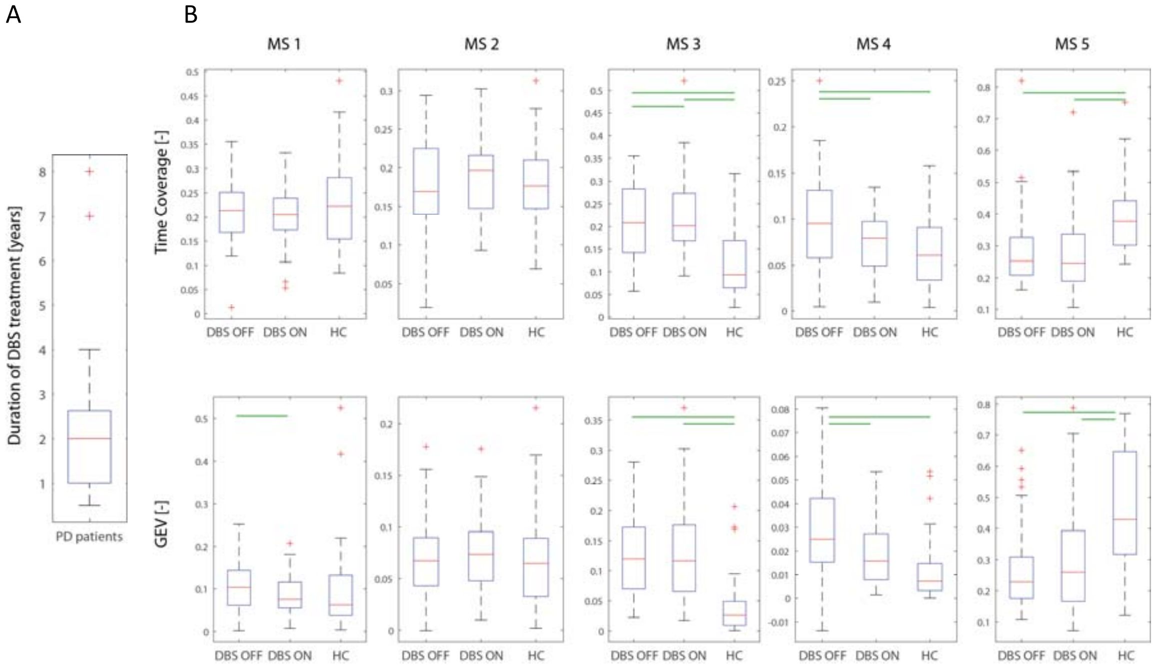

Supplementary Figure 3: A – duration of DBS treatment in PD patients. B - comparison of temporal parameters (time coverage top, GEV bottom) of five identified microstates in each analyzed group. The effect of DBS treatment duration was regressed out from the parameters in the DBS OFF and ON groups. Each box covers the data from 25th to 75th percentiles; the red line in each box represents the median over subjects in a particular group, and whiskers represent 1.5 times the interquartile range (IQR). Red crosses show the outliers. Green lines mark significant differences ($p < 0.05$ FDR).

EEG microstates – temporal parameters (mean duration and occurrence)

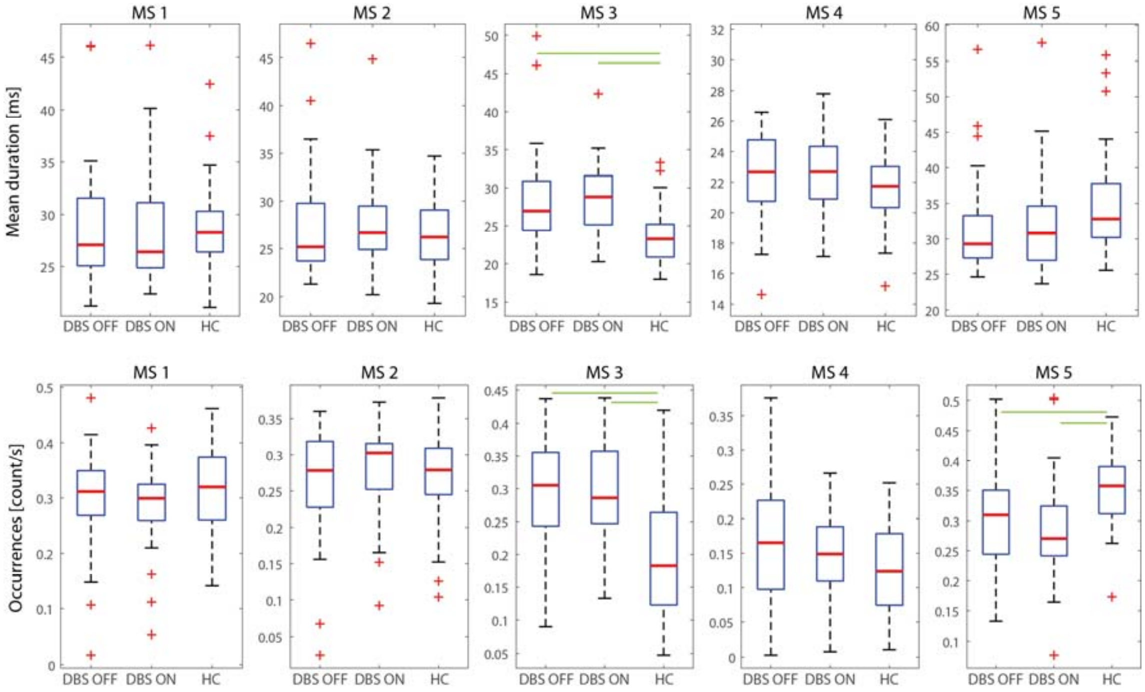

Supplementary Figure 4: Comparison of temporal parameters (mean duration top, occurrence bottom) of five identified microstates in each analyzed group. Each box covers the data from 25th to 75th percentiles; the red line in each box represents the median over subjects in a particular group, and whiskers represent 1.5 times the interquartile range (IQR). Red crosses show the outliers. Green lines mark significant differences (p < 0.05 FDR).

EEG microstates – spectral analysis of microstates

A – delta band

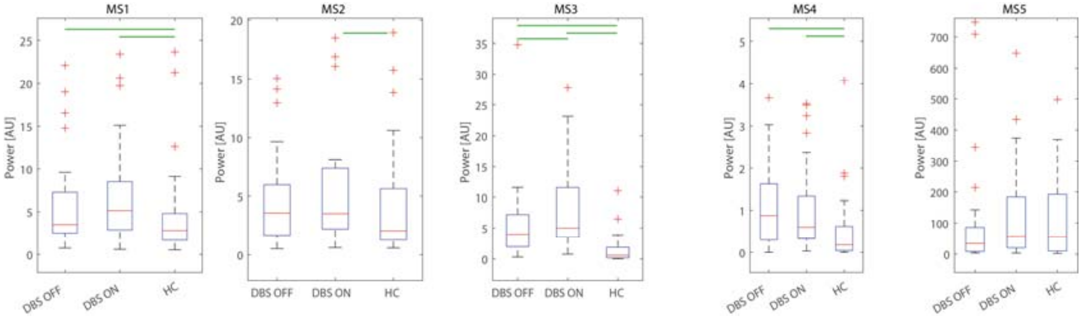

B – theta band

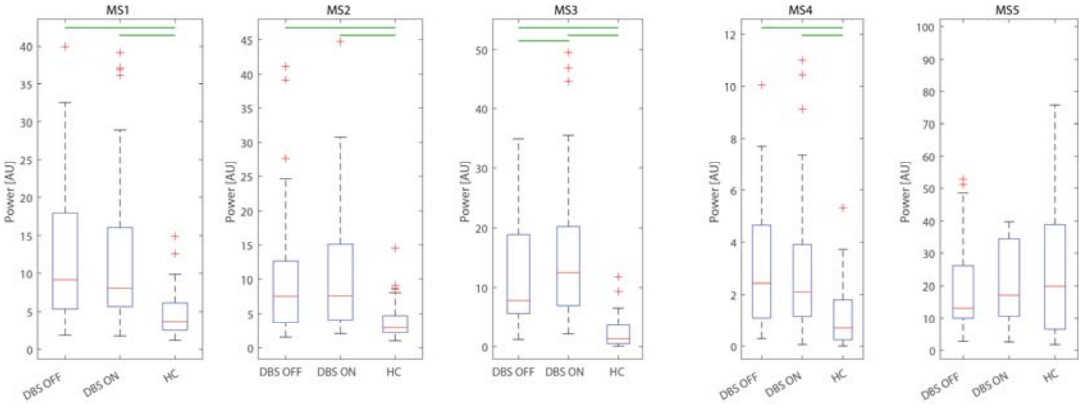

C – alpha band

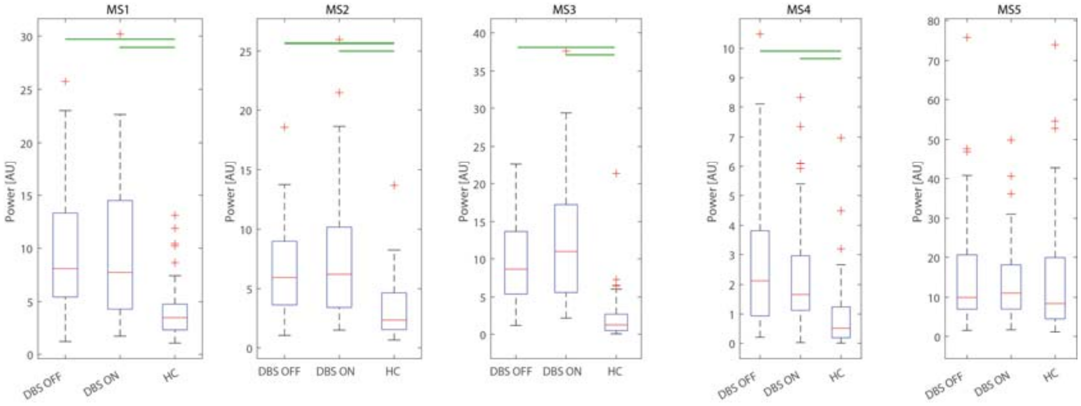

D – beta1 band

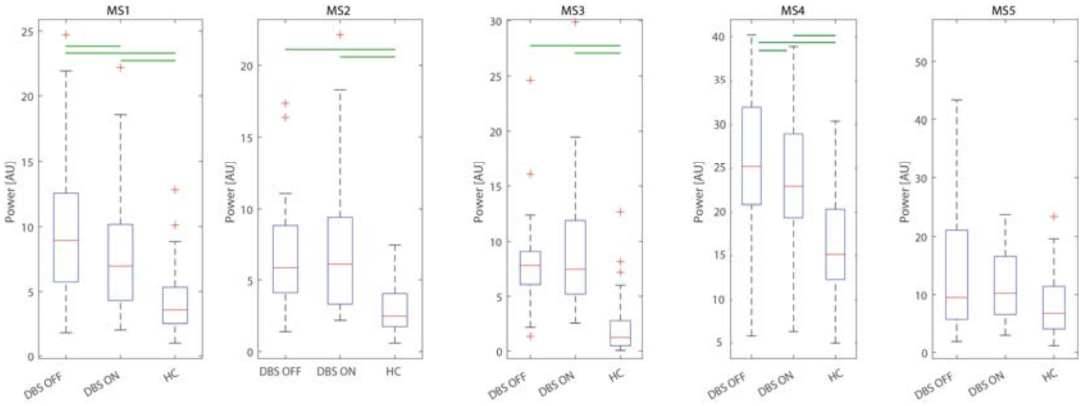

E – beta2 band

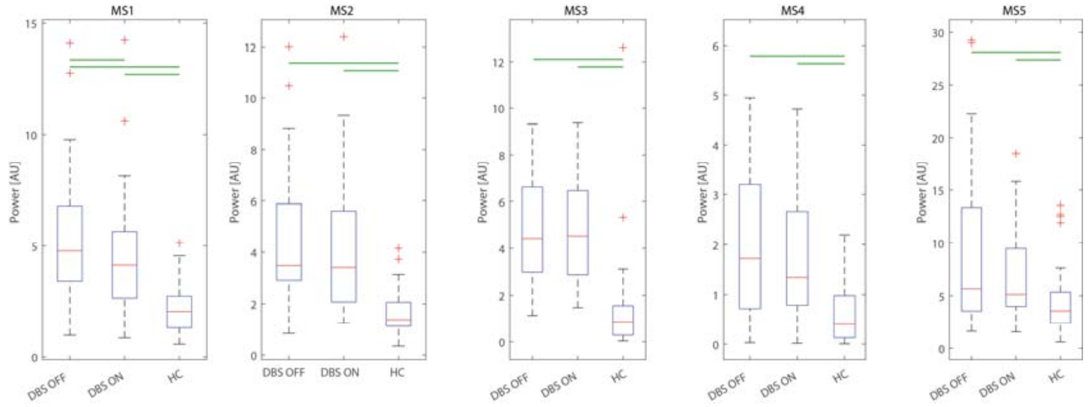

Supplementary Figure 5: Comparison of mean power (A – delta, B – theta, C – alpha, D – beta1, E – beta2 band) during the presence of five identified microstates in each analyzed group. Each box covers the data from 25th to 75th percentiles; the red line in each box represents the median over subjects in a particular group, and whiskers represent 1.5 times the interquartile range (IQR). Red crosses show the outliers. Green lines mark significant differences ($p < 0.05$ FDR).

EEG spectral analysis

A

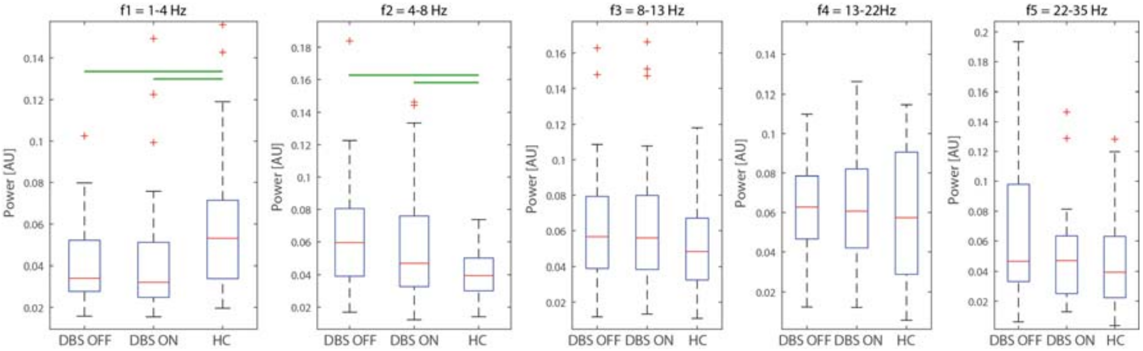

B

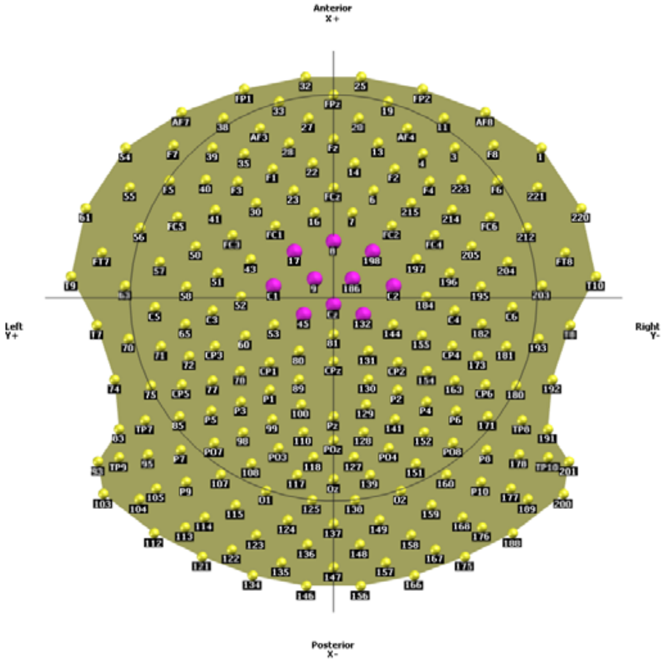

C

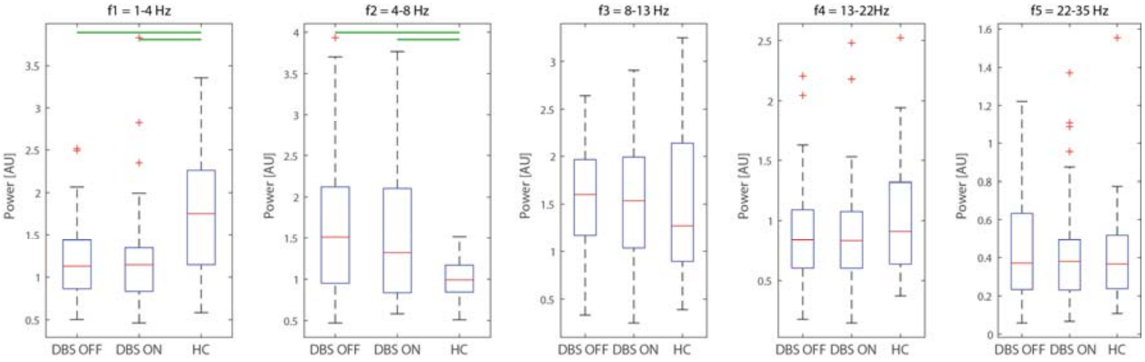

Supplementary Figure 6: Mean power from 5-minute sessions in each analyzed group. A – average of 204 channels, C – average of 10 channels (shown on B). Each box covers the data from 25th to 75th percentiles; the red line in each box represents the median over subjects in a particular group, and whiskers represent 1.5 times the interquartile range (IQR). Red crosses show the outliers. Green lines mark significant differences ($p < 0.05$ FDR).

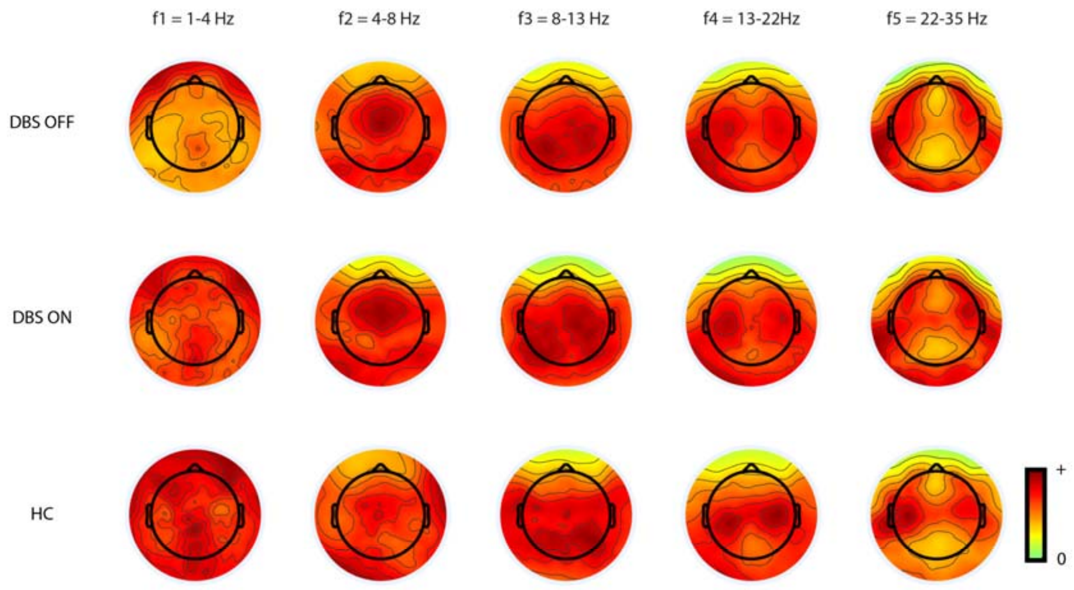

Supplementary Figure 7: Power spectral maps for five frequency bands analyzed from 5-minute sessions in each subject group

Electrical source imaging of microstates

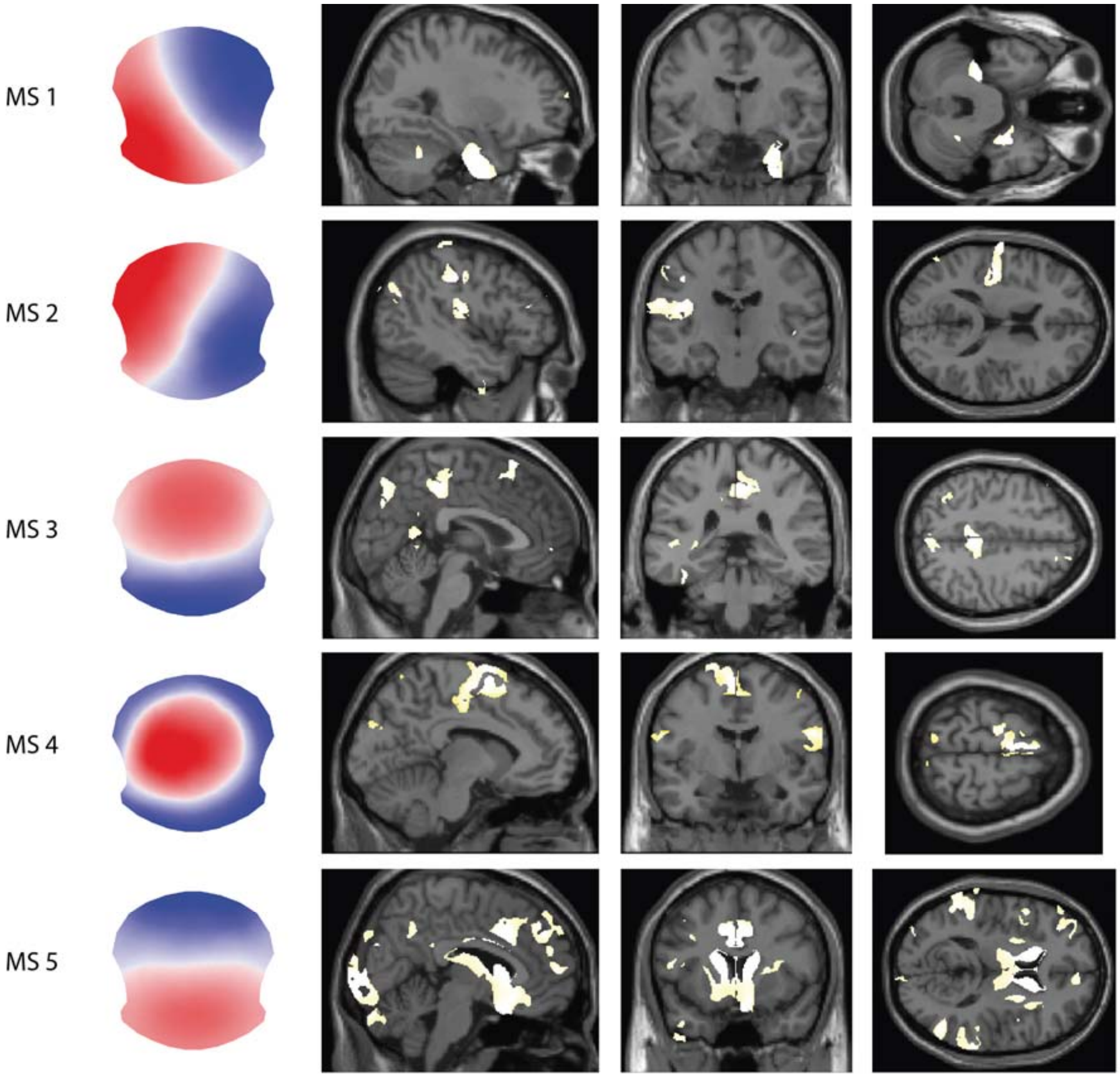

Supplementary Figure 8: Electrical source imaging of EEG segments where a particular microstate was presented (10% highest activations). Left – topography of revealed microstates, right – orthogonal slices of T1 MRI template with localized microstate sources.
